# Occupational asbestos exposure and urinary bladder cancer: a systematic review and meta-analysis

**DOI:** 10.1007/s00345-023-04327-w

**Published:** 2023-02-27

**Authors:** Nicolò Franco, Alessandro Godono, Marco Clari, Catalina Ciocan, Carlotta Zunarelli, Enrico Pira, Paolo Boffetta

**Affiliations:** 1 Department of Public Health and Pediatrics, University of Torino, Turin, Italy; 2 Department of Medical and Surgical Sciences, University of Bologna, Bologna, Italy; 3 Stony Brook Cancer Center, Stony Brook University, Stony Brook, NY USA

**Keywords:** Asbestos, Occupational health, Urinary bladder cancer, Systematic review

## Abstract

**Purpose:**

There is conflicting evidence on the association between asbestos exposure and bladder cancer. We performed a systematic review and meta-analysis to provide evidence on occupational asbestos exposure and the risk of mortality and incidence of bladder cancer.

**Methods:**

We searched three relevant electronic databases (Pubmed, Scopus, and Embase) from inception to October 2021. The methodological quality of included articles was evaluated using the US National Institutes of Health tool. Standardized incidence ratios (SIRs) and standardized mortality ratios (SMRs) for bladder cancer, as well as respective 95% confidence intervals (CIs), were extracted or calculated for each included cohort. Main and subgroup meta-analyses according to first year of employment, industry, sex, asbestos type, and geographic region were performed.

**Results:**

Fifty-nine publications comprising 60 cohorts were included. Bladder cancer incidence and mortality were not significantly associated with occupational asbestos exposure (pooled SIR: 1.04, 95% CI: 0.95–1.13, *P* = 0.000; pooled SMR: 1.06, 95% CI: 0.96–1.17, *P* = 0.031). Bladder cancer incidence was higher among workers employed between 1908 and 1940 (SIR: 1.15, 95% CI: 1.01–1.31). Mortality was elevated in asbestos workers cohorts (SMR: 1.12, 95% CI: 1.06–1.30) and in the subgroup analysis for women (SMR: 1.83, 95% CI: 1.22–2.75). No association was found between asbestos types and bladder cancer incidence or mortality. We observed no difference in the subgroup analysis for countries and no direct publication bias evidence.

**Conclusion:**

There is evidence that workers with occupational asbestos exposure have a bladder cancer incidence and mortality similar to the general population.

**Supplementary Information:**

The online version contains supplementary material available at 10.1007/s00345-023-04327-w.

## Introduction

The term asbestos comprises a group of natural minerals that form long, thin fibers when they crystallize [1]. Asbestos fibers tend to possess good strength properties (e.g. high tensile strength, wear and friction characteristics), flexibility (e.g. the ability to be woven), excellent thermal properties (e.g. heat stability and thermal, electrical and acoustic insulation), absorption capacity and resistance to chemical, thermal and biological degradation. Owing to its properties asbestos has been widely used worldwide and the story of this mineral was one of progressive commercial success until the mid-twentieth century [2]. The range of applications in which asbestos has been used includes roofing, thermal and electrical insulation, concrete pipes and sheets, flooring, gaskets, friction materials, coating and compounds, plastics, textiles, paper, mastics, thread, fiber jointing, and millboard. As the health risks associated with asbestos became increasingly recognized, its use began to decline. Despite widespread knowledge of the hazards of asbestos and bans on any use of asbestos in many countries, an estimated 1 million tons of this mineral was used around the world in 2020 [3].

In addition to the well-known association of asbestos with mesothelioma [4] and lung cancer [5], asbestos minerals have also been associated with ovarian [6], laryngeal [7] and gastrointestinal tract cancers [8]. Regarding other cancer sites, some epidemiological studies have reported an association between occupational exposure to asbestos and increased incidence of and mortality from bladder cancer [9–11]. Furthermore, asbestos fibers have been detected in tissue samples of bladder cancer patients affected by pulmonary asbestosis [12].

To our knowledge, no previous systematic reviews have been conducted on occupational asbestos exposure and the risk of mortality and incidence of bladder cancer. Thus, we aimed to perform a systematic review and meta-analysis to investigate this association.

## Materials and methods

This systematic review and meta-analysis was performed and reported according to the Preferred Reporting Items for Systematic Reviews and Meta-Analyses (PRISMA) guidelines [13].

### Search strategy

Three different databases were searched: Medline (PubMed), Scopus and Ovid (Embase). Firstly, a comprehensive search strategy was created using the following terms: "Neoplasms”, “Carcinoma”, “Asbestos”, “Amosite", “Crocidolite”, “Amphibole”, “Serpentine”, “Asbestosis". Next, a specific search strategy was performed adding the term “Bladder”. Results were restricted to studies conducted on humans, while no limits were applied for language. The databases were searched from inception to October 20, 2020. The resulting papers were hand screened and relevant references were evaluated to find any further relevant papers. Database searches were conducted with the aid of an expert librarian to ensure completeness and rigor. The search strategies for each database are given in Online Information 1.

### Inclusion and exclusion criteria

Only full articles published in peer-reviewed journals were considered. Cohort-studies and nested case-control studies of workers with a respiratory occupational exposure to asbestos in all industries and occupations were included. Cohorts of workers who were exposed to asbestos through ingestion only and had doubtful exposure to asbestos were excluded. We included all studies conducted on workers employed in industries or occupations in which asbestos exposure was considered substantial, such as asbestos workers, miners and millers, and shipyards. For studies of workers for whom occupational exposure to asbestos was possible (e.g. electricity workers or chimney sweeps [10, 14]), we used the criterion of a standardized mortality ratio (SMR) or standardized incidence ratio (SIR) for mesothelioma > 2 as marker of significant exposure, leading to the retention of the study for the meta-analysis. Descriptive studies, other systematic reviews or meta-analyses, community-based studies (either of cohort or case-control design), as well as conference proceedings, theses, and letters to the editor were excluded. Articles for which the full text was not available either online or by direct request to the journal in which they were published were also excluded. Two reviewers independently assessed the papers against the inclusion and exclusion criteria. Disagreement was solved by discussion.

### Data extraction

For each mortality study we extracted the SMR for bladder cancer and its 95% confidence interval (CI); when these measures were not directly available from publications, but raw data were reported, we calculated them. Similarly, we abstracted, or calculated if not specified in the text, the SIR and its corresponding CI for cancer incidence studies. If 90% CI was reported, it was converted to 95% CI. Results of internal analysis (e.g., based on hazard ratios) were used for dose-response. In cases of multiple reports from the same cohort, the most comprehensive results (i.e. those based on the largest number of cases) were used.

The following study characteristics were also extracted where available: publication year, study design, country, cohort size (or number of cases and controls), number of person-years, duration of employment, duration of follow-up, minimum time of exposure, duration of exposure, type of outcome (incidence or mortality). Data were extracted independently by two reviewers and any disagreement was solved by a third reviewer.

### Quality assessment

The methodological quality of the included studies was assessed through the National Institute of Health quality assessment tool for each study design [15]. The tools evaluate the presence of potential sources of bias, confounding factors, study power, and the strength of the association between the exposure and the outcome. The quality of the articles was rated as poor (score <9), fair (score = 9) and good (score >9). Quality assessment was performed by two independent reviewers, and results were discussed with the other authors until reaching consensus.

### Statistical analysis

The main analysis included results for ever vs. never asbestos exposure. Results across studies were combined, separately for studies of bladder cancer incidence and mortality, using random-effect models meta-analyses [16] based on the log-transformation of the SMR/SIR and its standard error. Inter-study heterogeneity was evaluated with the I2 test [17]. Stratified meta-analyses were conducted to explore potential sources of heterogeneity, by sex (>90% males, >90% females and <90% for both), period of first employment of cohort members (1908–1940, 1941–1949 and 1950–1993), type of asbestos (amphiboles, chrysotile, mixed and unspecified type), geographic region (Europe, UK, North America, Australia and Asia), and quality score (poor, fair and good). Finally, we assessed the presence of publication bias by visual inspection of the funnel plot and applying the test proposed by Egger et al. [18]. A meta-regression was performed to assess the association between the duration of asbestos exposure and bladder cancer. For studies reporting multiple SMR or SIR for different durations of exposure, a single meta-regression was performed and the results were then meta-analyzed.

## Results

A total of 13,267 articles were retrieved from Medline, Scopus, and Embase databases. After reviewing the titles, 2948 articles were considered potentially relevant, and after duplicates were removed, 2379 articles remained. Of these, 1639 articles were discarded following a review of the abstract. The full texts of the remaining 740 articles were examined in detail and assessed against the inclusion and exclusion criteria; 643 did not meet the inclusion criteria as described, and the full text was not available for a further 38. A manual search of the reference lists of included articles did not reveal any additional pertinent studies. Thus, 59 articles [9–11, 14, 19–73] met the inclusion criteria for the systematic review (Online Information 2).

The number of workers in each cohort ranged from 88 [65] to 160,640 [41], the number of incidences of bladder cancer per cohort varied from four [45] to 1257 [14], and that of bladder cancer deaths from zero [65] to 310 [9]. Bladder cancer incidence was evaluated in 26 cohorts, bladder cancer mortality was evaluated in 38, and both outcomes were reported in four cohorts [23, 24, 29, 63, 64]. The total number of bladder cancer cases and deaths across all articles was 3596 (out of 525,585 subjects) and 1169 (out of 385,552 subjects), with a follow-up duration ranging from five [59] to 88 years [10]. Selected characteristics of the cohorts are reported in Table 1.

**Table 1:** Characteristics of the included cohorts

Author(s), year	Country	Study design	Follow-up period	First year of employment	Type of Worker	Type of asbestos	No. of male subjects	No. of female subjects	Outcome studied	Quality assessment
Clemmesen and Hjalgrim-Jensen 1981 [28]	Europe	Cohort	1943–1976	1944	Asbestos workers	Mixed	5686	–	I	10
Acheson et al. 1984 [19]	UK	Cohort	1945–1980	1945	Insulation	Amosite	4820	–	M	9
Seidman et al. 1986 [62]		Cohort	1946–1982	1941	Asbestos workers	Amosite	820	–	M	8
Enterline et al. 1987 [31]	US	Cohort	1941–1980	1920	Asbestos workers	Chrysotile	1074	-	M	8
Hughes et al. 1987 [35]	US	Cohort	– 1982	1940	Asbestos workers	Chrysotile	5492	–	M	11
Sanden and Jarvholm 1987 [59]	Europe	Cohort	1978–1983	1950	Shipyards	Mixed	3787	–	I	7
Michaels and Zoloth 1988 [46]	US	Cohort	1976–1983	1953	Sheet metal workers	NS	331	–	M	8
Raffn et al. 1989 [54]	Europe	Cohort	1943–1984	1928	Asbestos workers	Chrysotile	7996	–	I	9
Selikoff and Seidman 1991 [61]	US	Case–control	1967–1986	1964	Insulation	NS	17,800	–	M	7
Simonato et al. 1991(i) [63]	Europe	Cohort	–	1946	Shipyards	NS	11,092	–	M	9
Simonato et al. 1991(ii)	Europe	Cohort	–	1946	Shipyards	NS	7626	–	I	8
Rapiti et al. 1992 (i) [56]	Europe	Subcohort	1965–1989	1936	Shipyards	NS	948	–	M	8
Rapiti et al. 1992 (ii)	Europe	Subcohort	1965–1989	1936	Shipyards	NS	1260	–	M	9
Danielsen et al. 1993 [30]	Europe	Cohort	1953–1990	1940	Shipyards	Chrysotile	4571	–	I	9
McDonald et al. 1993 [42]	Canada	Cohort	1976–1989	1950	Asbestos miners	Chrysotile	5351	–	M	9
Meurman et al. 1994 [45]	Europe	Cohort	1953–1991	1953	Asbestos miners	Antophyllite	736	–	I	10
Nokso-Koivisto and Pukkala 1994 [48]	Europe	Cohort	1953–1991	1953	Locomotive drivers	Mixed	8391	–	I	9
Johansen and Olsen 1998 [36]	Europe	Cohort	1968–1993	1908	Utility workers	NS	26,135	–	M	8
Tulchinsky et al. 1999 [67]	Asia	Cohort	1978–1992	-	Asbestos workers	Chrysotile	3057	–	I	10
Pira et al. 1999 [51]	Europe	Cohort	1950–1990	1950	Geothermal powerplant workers	Crocidolite	4237	–	M	11
Ronneberg et al. 1999 [58]	Europe	Subcohort	1953–1993	1946	Aluminum smelter workers	NS	2647	–	I	9
Langseth and Andersen 2000 [39]	Europe	Cohort	1953–1993	1920	Pulp and paper workers	NS	23,718	–	I	9
Berry et al. 2000 [25]	UK	Cohort	1951–1980	1933	Insulation	Mixed	4400	700	M	10
Band et al. 2001 [22]	Canada	Cohort	1969–1992	1950	Pulp and paper mill workers	NS	28,278	–	I	9
Ulvestad et al. 2002 [68]	Europe	Cohort	1953–1999	1942	Asbestos workers	Chrysotile	541	–	I	11
Koskinen et al. 2003 [37]	Europe	Cohort	1991–1998	1971	Asbestos workers	NS	23,285	–	I	8
Rafnsson and Sulem 2003 [55]	Europe	Cohort	1955–1998	–	Marine engineers	NS	6603	–	I	9
Finkelstein and Verma 2004 [33]	Canada	Cohort	1950–1999	1949	Pipe trades workers	NS	25,285	–	M	8
Ulvestad et al. 2004 [69]	Europe	Cohort	1953–1999	1930	Insulation	NS	1116	–	I	9
Habib et al. 2005 [34]	Australia	Cohort	1972–1998	1957	Nuclear industry workers	NS	3402	1315	M	8
Nichols and Sorahan 2005 [47]	UK	Cohort	1973–2002	1926	Electricity generation and transmission workers	NS	72,889	–	M	9
Parducci et al. 2005 [49]	Europe	Cohort	1960–2002	1960	Cigarette factory workers	NS	756	1585	M	8
Wilczyńska et al. 2005 [72]	Europe	Cohort	1945–1999	1945	Asbestos workers	NS	4497	–	M	9
Richardson et al. 2007 [57]	US	Subcohort	1950–2002	1950	Nuclear workers	NS	15,264	3619	M	11
Tsai et al. 2007 [66]	US	Cohort	1948–2003	1948	Petrolchemical workers	NS	9764	857	M	10
Sorahan 2007 (i) [64]	UK	Subcohort	1951–2003	1946	Refinery workers	NS	28,555	–	M	9
Sorahan 2007 (ii)	UK	Subcohort	1971–2003	1946	Refinery workers	NS	28,555	–	I	9
Bertolotti et al. 2008 [26]	Europe	Cohort	1950–2003	1912	Asbestos workers	Mixed	2657	777	M	10
Pesch et al. 2010 [50]	Europe	Cohort	1993–2007	1993	Asbestos workers	NS	576	–	M	9
Menegozzo et al. 2011 [43]	Europe	Cohort	1965–2005	1950	Asbestos workers	Mixed	1247	–	M	10
Tomioka et al. 2011 (i) [65]	Asia	Subcohort	1947–2007	1947	Shipyards	NS	88	–	M	10
Tomioka et al. 2011 (ii)	Asia	Subcohort	1947–2007	1947	Shipyards	NS	156	–	M	10
Bonneterre et al. 2012 [27]	Europe	Cohort	1979–2002	1936	Chlorochemical plant workers	NS	2742	–	I	9
Richardson et al. 2013 [11]	US	Subcohort	1960–2008	1943	Former nuclear workers	NS	3624		M	9
Andersson et al. 2013 [21]	Europe	Cohort	1958–2001	1939	Pulp and paper mill workers	NS	18,113	2292	I	10
Hogstedt et al. 2013 [10]	Europe	Cohort	1958–2006	1918	Chimney sweeps	NS	6320	–	I	9
Wang et al. 2013 [71]	Asia	Cohort	1972–2008	–	Asbestos workers	Chrysotile	586	279	M	11
Daniels et al. 2014 [29]	US	Cohort	1950–2009	–	Firefighters	NS	29,002	991	M	9
Daniels et al. 2014 [29]	US	Cohort	1985–1009	–	FIrefighters	NS	23,646	807	I	8
Wu et al. 2014 [73]	Asia	Cohort	1985–2008	1975	Shipyards	NS	4155	–	I	12
Allen et al. 2015 [20]	US	Cohort	1988–2010	1937	Taconite mine workers	NS	37,755	2953	I	9
Lin et al. 2015 [41]	Asia	Cohort	1980–2009	1950	Asbestos workers	NS	121,883	–	I	9
Van der Borre and Deboosere 2015 [70]	Europe	Subcohort	2001–2009	–	Asbestos workers	NS	2056	–	M	9
Levin et al. 2016 [40]	US	Cohort	–	–	Asbestos workers	Amosite	1121	9	M	10
Pira et al. 2016 [52]	Europe	Cohort	1946–2013	1946	Asbestos workers	NS	894	1083	M	11
Ferrante et al. 2017 [9]	Europe	Multiple Cohorts	1970–2010	1949	Asbestos workers	NS	46,060	5741	M	9
Pira et al. 2017 [53]	Europe	Cohort	1946–2014	1930	Asbestos miners	Chrysotile	1056	–	M	11
Barbiero et al. 2018 (a) [23]	Europe	Cohort	1989–2011	1974	Asbestos workers	NS	2488	–	M	9
Barbiero et al. 2018 (b) [29]	Europe	Cohort	1995–2009	–	Asbestos workers	NS	2488	–	I	8
Merlo et al. 2018 [44]	Europe	Cohort	1981–2014	1960	Shipyards	NS	3984	–	M	9
Schnatter et al. 2019 [60]	Canada	Cohort	1964–2006	1964	Petroleum workers	NS	19,942	9437	M	8
Sorahan 2019 [14]	UK	Cohort	1973–2015	1973	Electricity generation and transmission workers	NS	71,185	10,431	I	9
Koutros et al. 2019 [38]	US	Cohort	1942–2011	1942	Acrylonitril workers	NS	20,270	5190	M	10
Fazzo et al. 2020 [32]	Europe	Cohort	1986–2018	1941	Asbestos workers	NS	177	–	M	10

*NS* not specified, *M* mortality, *I* incidence

### Meta-analysis

**Fig. 1:**
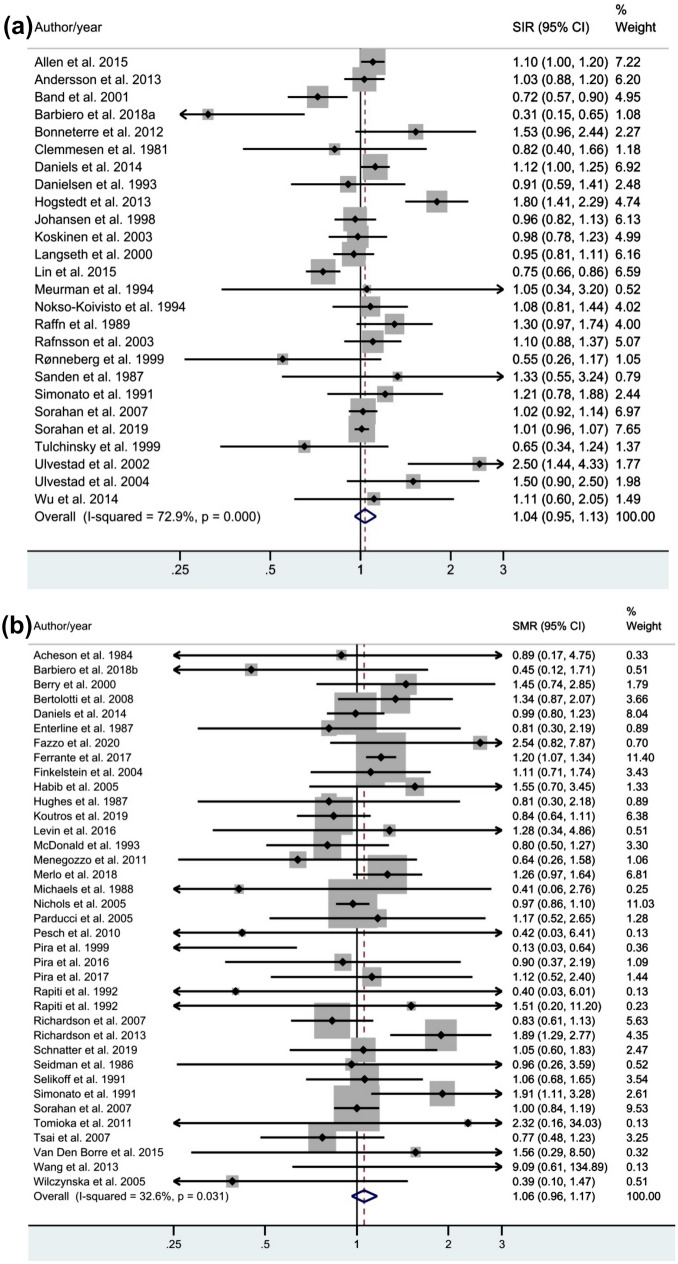
**a** Forest plot of the pooled standardized incidence ratio and 95% confidence intervals of urinary bladder cancer incidence associated with occupational asbestos exposure, using random-effect models. **b** Forest plot of the pooled standardized mortality ratio and 95% confidence intervals of urinary bladder cancer mortality associated with occupational asbestos exposure, using random-effect models

The results of the random-effects meta-analysis showed that bladder cancer incidence and mortality were not associated with occupational asbestos exposure. Results are shown in Figure 1a and b respectively (pooled SIR: 1.04, 95% CI: 0.95–1.13, *P*=0.000; pooled SMR: 1.06, 95% CI: 0.96–1.17, *P*=0.031). Both the pooled SIR and the pooled SMR showed significant heterogeneity (*P*<=0.001; *I2*=72.9%; *P*=0.031; *I2*=32.6%, respectively). One of the two cohorts analyzed in Tomioka et al. 2011 [65] could not be included in the meta-analysis because it reported no bladder cancer deaths (SMR: 0.00, 95%CI: 0.00–15.50).

Subgroup analysis stratified by first year of employment, industry, asbestos type, and geographic region showed that bladder cancer incidence increased significantly among workers employed between 1908 and 1940 (SIR: 1.15, 95% CI: 1.01–1.313) and bladder cancer mortality increased among women (SMR: 1.83, 95% CI: 1.22–2.75) and asbestos workers (SMR: 1.12, 95% CI: 1.06–1.30) (Table 2)

**Table 2:** Pooled SMR and SIR subgroup meta-analysis

Subgroup items	SMR Pooled results (95% CI)	Number of studies	Heterogeneity (*I*^2^), %	SIR Pooled results (95% CI)	Number of studies	Heterogeneity (*I*^2^), %
First year of employment						
1908–1940	1.00 (0.89–1.12)	8	0.0	1.15 (1.01–1.31)	9	71.5
1941–1949	1.11 (0.93–1.32)	12	53.6	1.12 (0.77–1.61)	5	70.4
1950–1993	1.00 (0.75–1.33)	11	41.9	0.90 (0.77–1.06)	7	74.6
Type of workers						
Textile workers	1.99 (0.23–17.06)	2	60.7	–	< 2	-
Shipyards	1.26 (0.97–1.63)	4	0.0	0.80 (0.46–1.41)	4	66.1
Insulation	1.35 (0.72–2.53)	2	0.0	–	< 2	-
Miners and millers	0.88 (0.59–1.30)	2	0.0	0.96 (0.79–1.17)	4	73.7
Asbestos Workers	1.12 (1.06–1.30)	13	0.0	1.04 (0.76–1.42)	6	82.2
Others	1.029 (0.89–1.19)	14	0.0	1.09 (0.99–1.19)	11	67.8
Sex						
Men only	1.06 (0.94–1.18)	30	35.1	1.03 (0.94–1.13)	25	73.1
Women only	1.83 (1.22–2.75)	4	0.0	–	< 2	–
Type of asbestos						
Amphiboles	0.86 (0.61–1.21)	4	54.1	–	< 2	–
Chrysotile	0.86 (0.29–2.52)	5	0.0	1.19 (0.75–1.90)	4	75.2
Mixed	1.21 (0.82–1.78)	3	16.3	1.06 (0.82–1.37)	3	0.0
Country						
Europe	1.16 (0.96–1.41)	16	28.2	1.11 (0.97–1.27)	18	67.7
UK	0.99 (0.90–1.09)	4	0.0	1.01 (0.96–1.06)2	2	0.0
US and Canada	0.98 (0.85–1.13)	14	23.7	0.99 (0.82–1.20)	3	84.1
Asia	4.58 (0.68–30.71)	2	0.0	0.76 (0.67–0.86)	3	0.0
Quality score						
Poor	1.07 (0.85–1.36)	9	0.0	0.96 (0.81–1.12)	8	70.2
Fair	1.08 (0.94–1.24)	17	59.9	1.07 (0.95–1.21)	11	82.2
Good	0.97 (0.80–1.17)	11	0.0	1.10 (0.86–1.41)	7	51.0

*SMR* standardized mortality ratio, *SIR* standardized incidence ratio, *CI* confidence interval

Meta-analysis of meta-regressions was possible for 5 studies. The RRs for each year of exposure were 0.97 (CI: 0.905–1.041) and 1.019 (CI: 0.997–1.041) for mortality and incidence respectively.

Funnel plots indicated no obvious outliers, and no evidence of publication bias was observed for either bladder cancer incidence or mortality. No small-study effect was found for mortality (*p* = 0.984) and incidence (*p* = 0.824) (Online Information 3).

## Discussion

We investigated the relation between occupational asbestos exposure and risk of bladder cancer with a meta-analysis of the results obtained from a wide systematic review. Incidence of and mortality from bladder cancer were not significantly increased among asbestos-exposed workers (pooled SIR: 1.04, 95% CI: 0.95–1.13, *P*=0.000; pooled SMR: 1.06, 95% CI: 0.96–1.17, *P*=0.031). These results were in line with the largest studies done on asbestos workers [14, 20, 21, 29, 47, 64], and were confirmed in subgroup analyses stratified by type of asbestos fibers, country and quality assessment.

Also, from a biological point of view, there is no evidence of a reasonable mechanism to elucidate how respiratory asbestos fibers could reach the bladder. In the literature, there are no established physio-pathological pathways nor pathological evidence that could explain increased bladder cancer incidence or mortality among workers exposed to respiratory asbestos fibers.

When stratifying by year of first exposure, an increased risk of bladder cancer was found for workers employed between 1908 and 1940. This result should be interpreted with caution because it derives from multiple stratified analyses.

Our results showed that female workers had a higher bladder cancer mortality rate. This result was based on five studies comprising a total of 27 observed deaths. The pooled result was greatly influenced by the article by Ferrante et al. [9], and was not confirmed in the parallel analysis of cancer incidence among women. Ferrante et al. reported that their preliminary analyses suggested that the risk of bladder cancer was concentrated in the industrial sectors where asbestos exposure was associated with combustion fumes and other agents related to metalworking and painting. These industries had been classified in relation to carcinogenic risk, including bladder cancer [74, 75]. Furthermore, in four out of five studies no data on smoking habits, the most important environmental risk factor for bladder cancer, were considered. Cigarette smoking prevalence is high among women working in the construction industry and in construction and extraction occupations, as shown in a study done by Mazurek et al. in the United States [76]. Also, the higher bladder cancer mortality in the asbestos workers sub-cohort can probably be attributed to the increased smoking habit rates in this subgroup compared to the general population, as shown in a recent study by Frost et al. [77].

Regarding the major influence of tobacco smoke in the etiopathogenesis of bladder cancer, results from a recent meta-analysis [78] delineate a pooled relative risk of bladder cancer disease-specific mortality of 1.47 (95% CI: 1.24–1.75) for all smokers. In line with this result, a combined analysis of 11 case-control studies from Europe shows that the proportion of bladder cancer cases among women attributable to ever smoking was 0.30 [79].

Furthermore, for other organs there is no evidence of a difference between men and women in the development of asbestos-related diseases. This datum could suggest that the excess in bladder cancer risk for women only is not likely to represent a causal association.

This systematic review and meta-analysis suffers from some limitations. Information about smoking habits of workers, which is the main risk factor for bladder cancer and a potential confounder, is lacking from the studies we reviewed. Thus, the pooled SMR and SIR could be overestimated.

Another limitation is the lack of quantitative data on asbestos exposure and duration of the employment in most cohorts. To analyze the dose-response effect we considered the duration of the employment as a proxy for the dose, but only 12 out 60 studies provided this datum. However, the meta-regression resulted in an absence of a dose-response effect.

## Conclusions

Our meta-analysis provides evidence that workers with occupational asbestos exposure have a bladder cancer incidence and mortality rate similar to the general population.

Excesses in bladder cancer risk in selected groups of workers, and in particular women, are not likely to represent causal associations; however, further studies are needed to evaluate whether female workers are more likely to develop bladder cancer when occupationally exposed to asbestos.

Due to the limited data on the duration of the exposure, it is not clear whether length of employment has a significant role in bladder cancer.

## Supplementary Information

Below is the link to the electronic supplementary material.

Supplementary file1 (PDF 385 KB)

Supplementary file2 (PDF 99 KB)

Supplementary file3 (PDF 142 KB)

## Data Availability

The data that support the findings of this study are available from the corresponding author, MC, upon reasonable request.
