# Supplementary material for: Occupational asbestos exposure and urinary bladder cancer: a systematic review and meta-analysis
Source: World J Urol. 2023 Feb 27;41(4):1005–15. doi: 10.1007/s00345-023-04327-w (PMC10159975; doi:10.1007/s00345-023-04327-w)
Supplement: Supplementary file 1 — Supplementary file1 (PDF 385 KB) [file 345_2023_4327_MOESM1_ESM.pdf]

Online Resource 2

Figure S2a Funnel plot of Egger's test to assess publication bias among studies included for the SIR meta-analysis

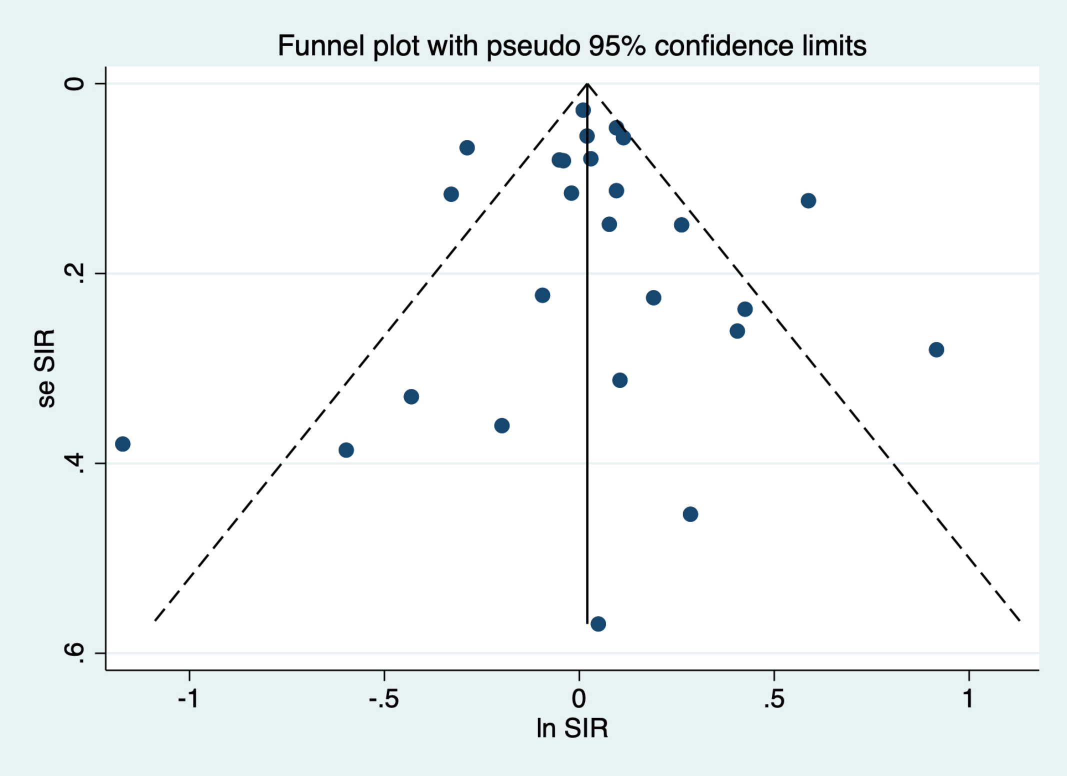

Figure S2b Funnel plot of Egger's test to assess publication bias among studies included for the SMR meta-analysis

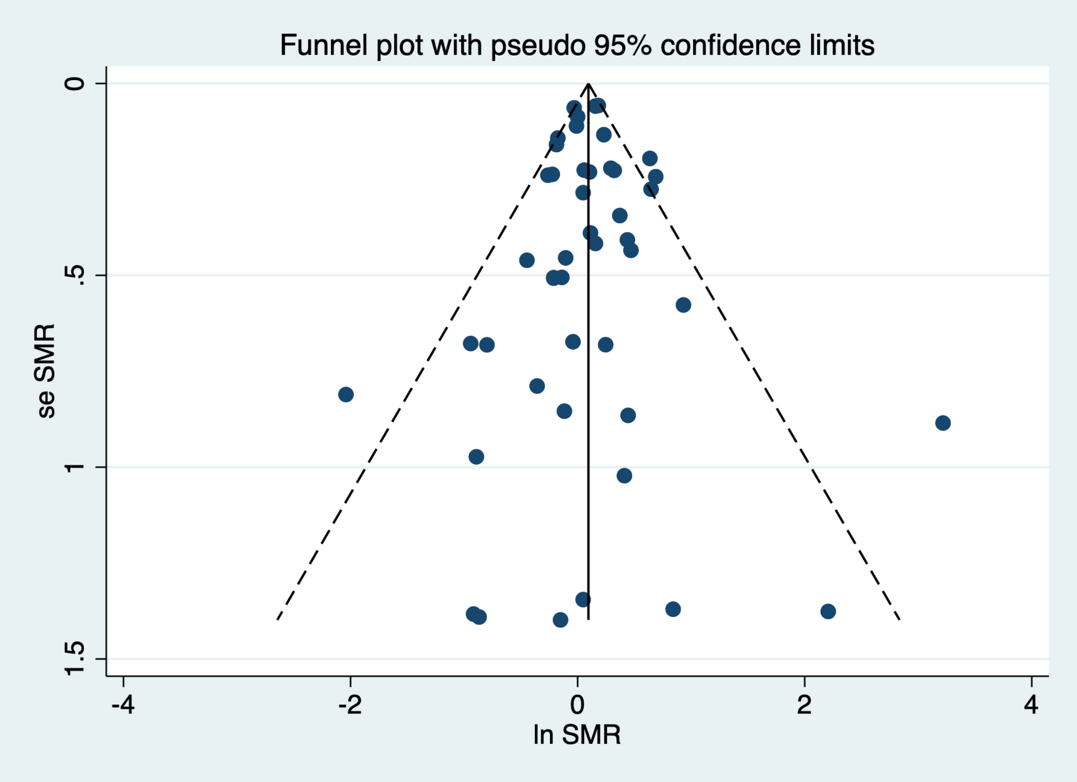

Occupational asbestos exposure and urinary bladder cancer: a systematic review and meta-analysis European Journal of Epidemiology, Franco et al. - Correspondence to Marco Clari
Department of Public Health and Pediatrics, University of Torino - marco.clari@unito.it
