# Supplementary material for: Occupational asbestos exposure and urinary bladder cancer: a systematic review and meta-analysis
Source: World J Urol. 2023 Feb 27;41(4):1005–15. doi: 10.1007/s00345-023-04327-w (PMC10159975; doi:10.1007/s00345-023-04327-w)
Supplement: Supplementary file 2 — Supplementary file2 (PDF 99 KB) [file 345_2023_4327_MOESM2_ESM.pdf]

Online Resource 1

Search strategies

General strategy

-Pubmed: ("Neoplasms"[Mesh] OR "Carcinoma"[Mesh]) AND ("Asbestos"[Mesh] OR "Asbestos, Amosite"[Mesh] OR "Asbestos, Crocidolite"[Mesh] OR "Asbestos, Amphibole"[Mesh] OR "Asbestos, Serpentine"[Mesh] OR "Asbestosis"[Mesh])

-Scopus: (TITLE-ABS-KEY (neoplasm* OR cancer* OR tumor* OR tumour* OR carcinoma) AND TITLE-ABS-KEY (asbestos OR asbestosis)) AND (LIMIT-TO (SUBJAREA , "MEDI") OR LIMIT-TO (SUBJAREA , "ENVI")) AND (EXCLUDE (EXACTKEYWORD , "Nonhuman") OR EXCLUDE (EXACTKEYWORD , "Animal")) AND (EXCLUDE (DOCTYPE , "le") OR EXCLUDE (DOCTYPE , "cp") OR EXCLUDE (DOCTYPE , "ed") OR EXCLUDE (DOCTYPE , "no") OR EXCLUDE (DOCTYPE , "ch") OR EXCLUDE (DOCTYPE , "er") OR EXCLUDE (DOCTYPE , "bk"))

-Embase: (asbestos:ti,ab,kw OR asbestosis:ti,ab,kw) AND (neoplasm:ti,ab,kw OR 'malignant neoplasm':ti,ab,kw) AND [humans]/lim

Specific strategy for bladder cancer

-Pubmed: (("Urinary Bladder"[Mesh]) OR (urothel*[Title/Abstract]) OR (bladder[Title/Abstract])) AND (("Asbestos"[Mesh]) OR (amosite[Title/Abstract]) OR (crocidolite[Title/Abstract]) OR (serpentine[Title/Abstract]) OR (chrysotile[Title/Abstract]) OR (amphibole[Title/Abstract]))

-Scopus: (TITLE-ABS-KEY (*bladder*) OR TITLE-ABS-KEY (*urothel**) OR TITLE-ABS-KEY (*urinar**)) AND (TITLE-ABS-KEY (*asbestos*) OR TITLE-ABS-KEY (*amosite*) OR TITLE-ABS-KEY (*chrysotile*) OR TITLE-ABS-KEY (*amphibole*) OR TITLE-ABS-KEY (*crocidolite*) OR TITLE-ABS-KEY (*serpentine*)) AND (LIMIT-TO (SRCTYPE , *"j"*)) AND (EXCLUDE (DOCTYPE , *"re"*) OR EXCLUDE (DOCTYPE , *"le"*) OR EXCLUDE (DOCTYPE , *"cp"*) OR EXCLUDE (DOCTYPE , *"no"*) OR EXCLUDE (DOCTYPE , *"ed"*))
-Embase: ('bladder disease':ab,ti OR 'bladder cancer':ab,ti) AND (asbestos:ab,ti OR asbestosis:ab,ti)

Department of Public Health and Pediatrics, University of Torino - marco.clari@unito.it
