# Supplementary figures and images for: Occupational asbestos exposure and urinary bladder cancer: a systematic review and meta-analysis
Source: World J Urol. 2023 Feb 27;41(4):1005–15. doi: 10.1007/s00345-023-04327-w (PMC10159975; doi:10.1007/s00345-023-04327-w)

## Prisma Diagram

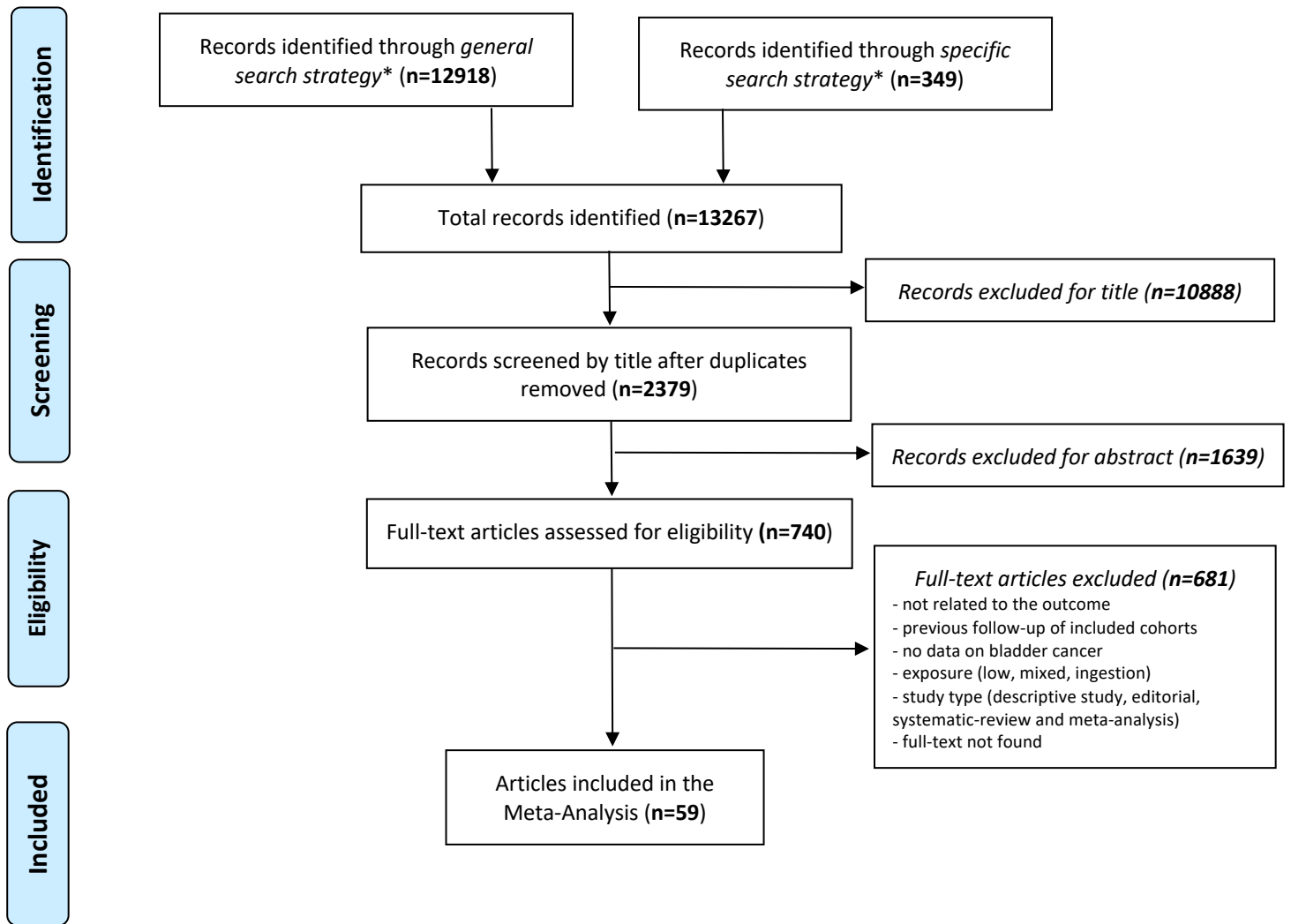

\* Specified in Supplementary Material 1

Supplement: Supplementary file 3 — Supplementary file3 (PDF 142 KB) [file 345_2023_4327_MOESM3_ESM.pdf]
